# A general methodology for collecting and preserving xystodesmid and other large millipedes for biodiversity research

**DOI:** 10.3897/BDJ.3.e5665

**Published:** 2015-08-17

**Authors:** Jackson C. Means, Elizabeth A. Francis, Avery A. Lane, Paul E. Marek

**Affiliations:** ‡ Virginia Tech, Blacksburg, United States of America; § University of Arizona, Tucson, United States of America

**Keywords:** millipede, detritus, mimicry, evolution, collection, α-taxonomy, DNA, RNA, specimen

## Abstract

**Background:**

With an estimated 80% of species remaining undescribed (but see Brewer et al. 2012), millipede taxonomy offers the opportunity to discover new species and explore biodiversity. The lack of basic alpha taxonomic information regarding millipedes belies their significant ecological role and potential as premier models in ecological and evolutionary studies. The group possesses many fascinating biological properties (*e.g.*, bioluminescence, mimicry, and complex chemical secretions) that have been the focus of several recent studies and are emerging avenues of future investigation.

**New information:**

Here we summarize a methodology for large-bodied millipede collection, curation, and preservation for genetic analyses with the hope that sharing these techniques will stimulate interest in these charismatic detritivores.

## Introduction

Millipedes perform an invaluable ecological service in the form of detritus fragmentation and nutrient recycling (Crawford 1992, Smit and Van Aarde 2001). They also serve as model organisms to address many evolutionary, ecological and biological concepts and questions. For example, recent research has exposed the existence of widespread mimicry rings of millipedes within the family Xystodesmidae (Marek and Bond 2009), bioluminescent aposematism (Marek et al. 2011), millipede-mite symbiotic interactions (Swafford and Bond 2009), long-held questions about arthropod trait evolution (e.g., the evolution of spiracles in insects and myriapods, Wesener et al. 2014), and ancient stem group relationships of arthropods (Shear and Edgecombe 2010). While there have been interesting studies on millipedes in recent years, they remain a largely unexplored group, with only ~12,000 of the predicted 60,000 (Brewer et al. 2012) to 80,000 (Hoffman et al. 2002) species currently described. Furthermore, relationships between described species are poorly understood, resulting in uncertainty in classification and an abundance of monotypic genera with unclear evolutionary affiliations (Sierwald and Bond 2007). Although fundamental questions of arthropod relationships have been established on the basis of classical *α*-taxonomic work using decades-old alcohol preserved collections, many research questions require an accurate evolutionary context established with molecular phylogenetics. These questions require fresh specimens and genetic material and therefore field collections are necessary for many areas of millipede research. Furthermore, the impact of habitat fragmentation and degradation have made collections-based research imperative to counteract the loss of species before their discovery (e.g. anonymous extinction) and to understand critically threatened biodiversity and its pivotal role in ecosystem function.

Several methods have been previously outlined for the collection of millipedes (Mesibov et al. 1995, Snyder et al. 2006). Of the methods compared (pit-fall traps, leaf litter examination with Berlese funnels, and hand collecting) hand collecting was found to be the most efficient, save perhaps for the highly specialized use of baited pit-fall traps in cave systems (Lewis and Pursell 1997, Schneider and Culver 2004). However, hand collection can be an inefficient and strenuous activity if unsuitable habitats are selected, and researchers may be in danger of physical harm when collecting by hand under logs, rocks or leaves, especially in tropical environments. Therefore xystodesmid and other large-bodied millipede sampling requires an elementary set of techniques to improve efficiency, reduce effort and remove investigators from unnecessary risk. Furthermore, to the authors’ knowledge, there currently exists no detailed methodology for hand collecting large-bodied millipedes, storing specimens live for photography, and processing tissue. Here we describe a simple protocol to aid in the collection of large millipedes, as well as a detailed description of millipede preservation, curation, and long-term storage of specimens, tissues, and genetic material. As these methods have been developed for xystodesmid millipedes, readers should take note that 'xystodesmid' in the follow text can only occasionally be generalized to other millipede groups and care should be taken in modifying methods for other taxa.

## Material and methods

The following methods have been developed for collecting xystodesmid millipedes in the Appalachian Mountains. They have also been successfully used throughout California and Costa Rica and may serve as a basis for collections-based taxonomic research in other litter-dwelling arthropod groups.

### Site Identification

With the exception of desert-dwelling species, millipedes generally lack a waxy epicuticle, rendering them permeable to water and susceptible to desiccation (Crawford 1979). Most species are therefore commonly found in dark, moist areas, typically occurring beneath leaves, logs or rocks (Cloudsley-Thompson 1950). Areas near water, with ample leaf accumulation and a thick forest canopy, are the most attractive to millipedes (Fig. 1). The mixed mesophytic deciduous forests of the Appalachian Mountains offer ideal conditions for xystodesmid millipedes and are a center of diversity for this family (Marek et al. 2014). This topographically rugged area possesses a variety of habitat types (*e.g.*, dark gullies, hollows, and stream corridors), and this has led to the separation of populations for long periods of time, promoting evolutionary divergence and speciation (Marek 2010). Sharp bends in mountain roads are often prime collection areas, as they are commonly located in gullies where leaves accumulate and moisture levels are elevated. Stinging nettle (*Urtica dioica*) and poison ivy (*Toxicodendron radicans*) also thrive in these areas of high moisture and may act as an indicator of xystodesmid millipede presence. Forest roads that have fallen into disuse are also excellent areas for millipede collection. For example, sunken roads and old wagon trails have yielded a diversity and abundance of millipedes. We have observed that the sides of gravel roads possess higher xystodesmid millipede abundance than on the forest floor, perhaps due to accumulation of moist leaf debris and a greater amount of calcium associated with certain types of rocks and gravel, such as limestone, used for road surfacing. Millipedes are also commonly encountered in karst and limestone rich regions for this reason. Based on an experiment to assess the effect of calcareous road materials on millipede and snail abundance in Appalachia, Kalisz and Powell (Kalisz and Powell 2003) found that snail and millipede dry mass was 10- and 3-fold higher at the roadside than at 50 m away. In general one can expect to find a lower diversity and abundance of millipedes in areas that are rarely disturbed, while moderately disturbed areas typically have a higher diversity and abundance (Connell 1978). This phenomenon is most noticeable while collecting millipedes in tropical old growth versus new growth forests. Regions with low disturbance may contain rare or endemic species and should be explored thoroughly even when low abundance makes collection difficult.

### Collecting Kit

**Figure 2.**
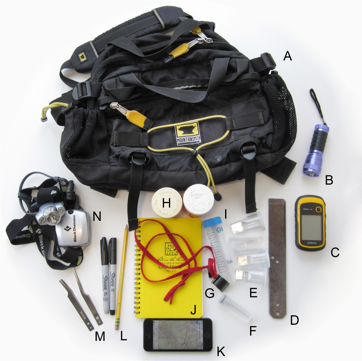
Field collection kit, including a (A) Mountainsmith lumbar pack (8 L volume); (B) UV flashlight; (C) GPS unit; (D) breaker bar; (E) 20 mL collection vials (cell counter type); (F) 100% alcohol-filled vials for millipedes that prematurely die (Sarstedt 8 mL plastic screw-top vials); (G) 10X and 20X Coddington loupes; (H) 100 mL collection vials (large pharmacy pill vial type); (I) 50 mL Falcon tube; (J) Field notebook; (K) Apple iPhone with internal GPS and Gaia GPS app with predownloaded USGS topo quads; (L) pencil, fine and extra fine point permanent Sharpie markers; (M) narrow and wide tip featherweight forceps from Bioquip; (N) headlamp from Black Diamond (Ion model).

**Figure 3.**
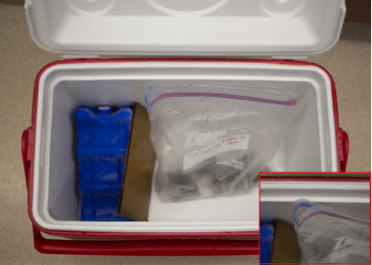
Cooler for transporting millipedes from the field to laboratory. An ice pack keeps the contents cool while a divider protects the samples from the ice. Inset shows a corner of the resealable bag containing the millipedes that has been opened to allow air transfer.

Our standard field collecting kit (Fig. 2), which can be packed in an Mountainsmith lumbar pack (Fig. 2A), includes a UV blacklight for nighttime collecting (Fig. 2B); a GPS unit with GLONASS to record latitude, longitude, and GPS elevation of specimen localities (Fig. 2C); a breaker bar for dismantling decaying logs (Fig. 2D); 20 mL collection vials (Fig. 2E); 100% ethanol for emergency preservation of millipedes that prematurely die (Fig. 2F); collection cards printed on archival paper to record locality details; 10X and 20X Coddington loupes (Fig. 2G); 100 mL collection vials (Fig. 2H); 50 mL Falcon tubes (Fig. 2I); Rite in the Rain field notebook (Fig. 2J); iPhone with internal GPS (or with external Bad Elf GPS plug-in), camera, and Gaia GPS app for navigation (Fig. 2K); Sharpie fine point and extra fine point permanent markers and a pencil (Fig. 2L); narrow and wide tip featherweight forceps from Bioquip (Fig. 2M); and a head lamp (Fig. 2N). A millipede rake is also included as part of the kit, and other items that are used once the collector starts field-processing material (typically at the car or campsite). These items include 1 L resealable bags to separate samples from different sites, water for keeping millipedes moist, and a cooler for millipede transport (Fig. 3). At all times, investigators are required to carry a collections permit, especially if collecting is to take place on national park land or other federally, state, or privately-owned property. In some cases, an additional permit is required to export specimens between countries or districts within the country.

### Millipede Rake

**Figure 4.**
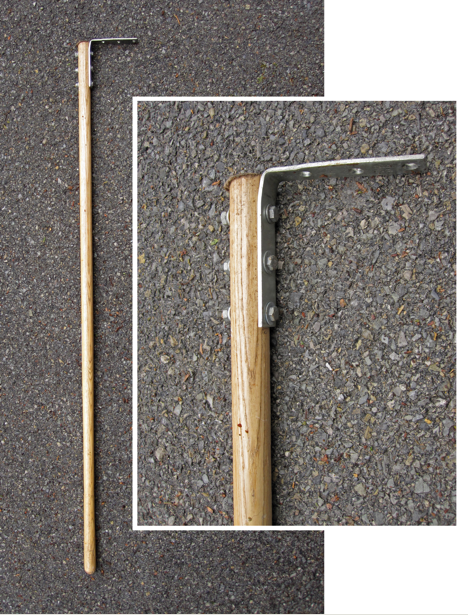
Millipede rake, consisting of a broom handle attached by nuts, bolts, and washers to a corner brace. Inset shows the rake head in detail.

Xystodesmid millipedes can often be found beneath leaf litter and as such a tool for the removal of litter is helpful in their collection. The millipede rake, originally adopted by Rowland Shelley (pers. comm.), consists of a wooden broom handle with a metal corner brace bolted to one end (Fig. 4). The flat metal end is used to scrape away leaves and debris and to lift small branches (Fig. 5). The rake should not be used to move heavy debris (> 3 kg), such as logs or rocks, as the tip can easily bend under high pressure. Debris should be returned to its original location to reduce desiccation and impact to habitat. Care must be taken to insure that the rake is scraped gently under debris as millipedes may be damaged when the rake is used with excessive force. Millipedes exposed by removal of leaf matter will typically curl into a tight ball for protection or attempt to flee. The relatively slow movement of millipedes (0.5 cm/s) makes their capture a simple procedure (Marek et al. 2011). The millipede rake may also be used to move snakes, clear a path through stinging nettle, or as a support when moving through difficult terrain.

### Other Useful Equipment

(i) UV flashlight (Fig. 2B). Many species of Polydesmida are fluorescent under blacklight, and a portable UV flashlight (400 nm peak wavelength) is an essential tool to collect millipedes of the family Xystodesmidae in California. These nocturnal animals are elusive during the day, but can be readily discovered at night with the aid of a portable blacklight, commonly used by geologists to find fluorescent rocks and minerals or by arachnologists to search for scorpions.

(ii) Gaia GPS app (Fig. 2K). While collecting abroad and in new areas, usage of high quality topographical maps is essential to pinpoint suitable habitats for millipedes. The iPhone and iPad app, Gaia GPS uses the built-in GPS receiver of the device (obviating the need for a Wi-Fi or a cellular signal) and pre-downloaded maps to plot the user’s position and bearing. An external GPS, such as a Bad Elf Plug-in (http://bad-elf.com) can improve GPS accuracy. Tools such as Gaia GPS and Bad Elf GPS plug-ins are helpful to navigate the roads of foreign countries that do not have road signs, as your position is plotted in real-time as you drive. An iPhone or iPad is also useful to capture pictures and movies and to store digital versions of collecting permits and relevant documents. The panorama camera setting is useful to document a 360° image of the habitat for the species record.

(iii) Breaker bar and headlamp (Fig. 2D,N). A breaker bar is convenient for prying and dismantling decaying logs and a headlamp is essential for examination of the woody debris for small millipedes.

(iv) Coddington loupes (Fig. 2G). Using a 20X Coddington loupe, species characteristic gonopods can be examined in the field. Grasp the male xystodesmid millipede with its head between the index and middle fingers and tail between the middle and ring fingers and move the 20X loupe about 5 mm from the gonopods. Gonopodal morphology, color pattern, and locality in combination will provide an accurate identification for nearly all xystodesmid millipedes on the planet. Small specimens < 20 mm in length often cannot be identified in this manner.

### Millipede Storage

**Figure 6.**
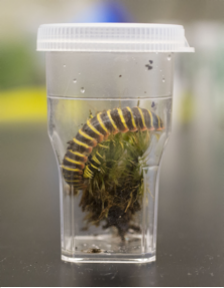
An example of a xystodesmid millipede, *Apheloria virginiensis corrugata* (Wood 1864) in a 20 mL collection vial with moist moss and detritus. (Also see Mov. 3.)

**Figure 8.**
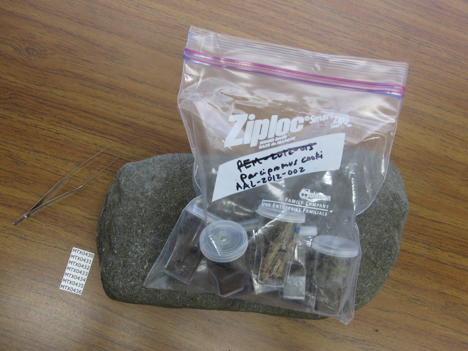
All vials from a single location should be kept in a single resealable bag, labeled with a collection code (e.g., AAL-2012-002) and opened slightly at the top for gas transfer.

Once collected, xystodesmids should be stored in 20 mL plastic cell-counter vials with a piece of moistened moss to reduce desiccation (Fig. 6, Fig. 7). For international shipping, a dampened Kimwipe (Kimberly-Clark, Roswell, GA) should replace moss or soil to comply with agricultural shipping regulations. Moisture level should be high enough that condensation is visible on the sides of the container, but not so much that water pools at the bottom of the vessel. Vials are 55 mm in height and 32 mm in diameter with a square base that is tapered. These vials are appropriate for many types of millipedes, though some genera such as *Narceus* and *Cleptoria* may require larger containers. A 400 mL jar (about the size used for peanut butter) is useful for these large-bodied specimens. When collecting, lids should be punctured with small holes to provide oxygen to the animals; however, holes should be punched prior to enclosing the millipede. Minuscule specimens (< 3 mm wide) may escape through these air holes, and therefore should be kept in airtight containers for transport back to the lab. Special care should be taken with airtight containers and the specimens should be aerated at least 2 – 3 times per day by opening the vial. When collecting in drier regions, for example the lowlands of California, fewer or no holes should be made in the lid to prevent desiccation. All vials from a single location should be kept in a single resealable bag, labeled with a collection code (e.g., HERP-002-2004) and opened slightly at the top for gas transfer (Fig. 3, Fig. 8). Bags containing samples should be stored in a cooler with an ice pack, and kept separate from direct contact with the ice (e.g., with a piece of cardboard or plastic, Fig. 3). This provides the millipedes with a cool, dark environment and reduces stress during transport. Xystodesmids on average will survive in a vial with moist moss for about a week, though processing (see below) should take place as soon as possible to avoid premature death and putrefaction. When processing does not take place immediately, the moisture level of the container, as well as the millipede’s condition, must be monitored daily. If a millipede is mortally injured in the field, and is unlikely to survive the trip back to the lab, then it should be placed in a vial containing 100% ethanol for DNA preservation. Otherwise, putrefaction nearly always causes complete loss of DNA.

### Collection Cards

**Figure 9.**
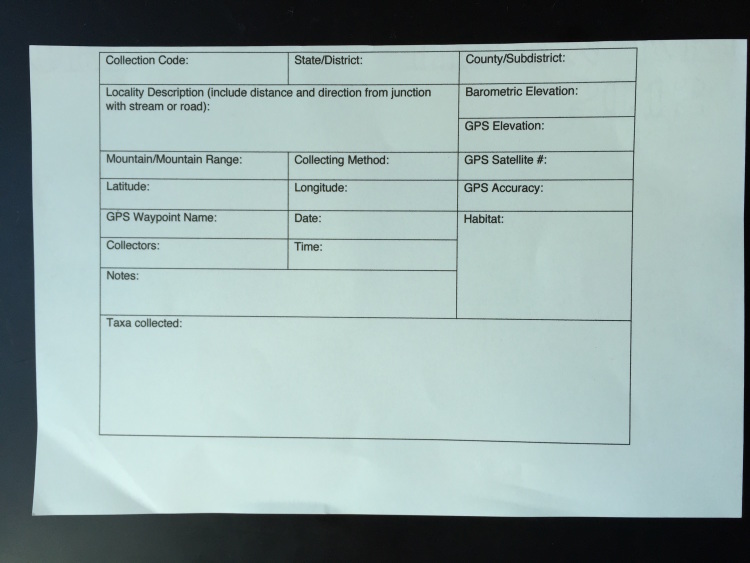
A collection card for recording geographical information in the field.

Supporting geographical information is essential for collections based biodiversity research and a specimen without a locality is nearly useless as a museum specimen. The location data recorded on collection cards (Fig. 9, Suppl. material 1) provides critical information about location, habitat, and the accuracy of GPS measurements. These data are essential for generating accurate specimen labels and understanding species distribution and how distributions may change over time. Additional information, such as leaf litter composition, moisture and light levels, and dominant flora can elucidate the ecological characteristics of the area. Collection cards should be printed with an inkjet printer on Resistall paper to ensure durability (e.g. an EPSON Stylus C88 with black DURABrite® pigment based ink). Each collection card is identified by a unique collection code (e.g., AAL-2012-002), which is then used to label the resealable bag thereby serving to identify the cohort of vials collected from an area during a collecting event. Other information recorded on the card includes (1) the state or district; (2) the county or subdistrict; (3) a description of the locality, specifically topography and proximity to junctions of roads or trails; (4) barometric and GPS elevations; (5) the number of satellites informing the GPS reading; (6) the satellite accuracy in meters; (7) the road name, mile marker and any nearby road junctions; (8) the mountain or mountain range in which the collection is taken; (9) the latitude and longitude recorded in decimal degrees, and a unique GPS waypoint name (ideally the collection code); (10) the date and time (in 24 hour format); (11) the unique codes given to any images taken; (12) a description of the habitat, specifically the dominant flora and tree species present; (13) all taxa collected and the numbers and gender of each; and (14) any natural history observations or notes.

### Rearing Immatures & Housing Live Specimens

Occasionally juvenile xystodesmid millipedes, which are soft and not yet fully sclerotized, must be collected due to possession of unique gonopods (indicating a new species or form) or an otherwise interesting biological feature. Juvenile specimens should be kept in the laboratory to develop into an adult. Developing xystodesmids should be stored in glass terrariums or 5 L battery jars with soil, moss and detritus from the habitat where the specimen was collected. As a result, millipedes that are not yet mature may complete their development for preparation and preservation later. Care should be taken not to disturb the specimens (*e.g.*, sifting through the leaves or persistently checking) because individuals that are molting are extremely delicate and the molting chambers in which millipedes undergo ecdysis are fragile and easily damaged.

### Live Photography

Millipedes representing new taxa, with unique color patterns, or lacking a photograph, should be documented while alive using a high resolution (≥ 10 megapixels) digital camera (*e.g.*, a Canon EOS 6D with a 65 mm macro lens for smaller genera ≤ 2 cm and a 50 mm macro lens for larger-bodied genera ≥ 2 cm). A flash with a diffuser should be used to produce soft light that will not generate glare (*e.g.*, a Canon Macro Twin Lite MT-24EX flash). Photographs should be captured from above (*dorsal habitus*) and from the side (*lateral habitus*) to record color patterns of the tergites, legs, and other external features. In case of millipedes with a color spot on the prozonite, an image of the millipede in a defensive coil should be captured. A bed of moss should be used to reduce light reflection and provide a natural background. Each photograph is assigned a unique code derived from the individual specimen code (e.g., if the specimen code is MPE0003 then the images should be named MPE0003_1, MPE0003_2…). These unique codes link image files with specimens, and are used to track and retrieve images in digital image archives.

### DNA & RNA Storage

After returning from the field with live specimens, material should be kept at room temperature (20°C), or in an environmental chamber maintained at 12°C and a light-to-dark setting of 12:12. The following protocol describes methods for the stabilization and storage of tissues for subsequent use in genetic analyses. Before beginning specimen processing, sterilize the bench area with a 10% bleach solution and gather required materials. Prepare one 1.5 mL microcentrifuge tube for each millipede by adding 500 µL of RNA*later* (Qiagen, Valencia, California) to each and placing in a tube rack. Use an alcohol resistant pen to record the specimen code on Tough Spots label stickers (USA Scientific, Ocala, Florida) for each of the tubes. Dumont 3C tweezers should be used to dissect legs for storage in RNA*later*. To sterilize between processing each specimen, dip the end of the tweezers into 100% ethanol, ignite with a flame, and hold upright until the flame is extinguished. Then, hold the tips of the tweezers in the lower blue region of the flame for 3 s, followed by a rinse of deionized water. Carefully place the sterilized tweezers on a fresh Kimwipe or weigh paper to maintain sterility before use. Fill specimen vials ¾ full (~ 10 mL) with 70% isopropanol. Take precaution to not let any fecal material, soil, parasitic mites, or other organic material contaminate the tissue collected for analysis. If needed, use a moistened Kimwipe (Kimberly-Clark, Roswell, GA) to wipe away debris from arthropods and gloves. Assign a unique specimen code label for each animal (already printed on Resistall paper) and record the code in pencil on the collection record. Grasping the xystodesmid ventral side up between the index and middle fingers and thumb, use the 3C tweezers to dissect legs at their base from the left posterior side of the millipede and remove legs anteriorly to segment 8 (the segment immediately posterior to the gonopods in males, see Fig. 10). Submerge the tissue in the microcentrifuge tube with RNA*later*. Millipede specimens should be preserved as museum specimens in shell vials (Fisher Scientific, Waltham, MA) containing 70% isopropanol with the printed specimen code reading left to right when the capped top is grasped in the right hand and turned clockwise. Prior to starting the next specimen, repeat the above flame-sterilization method and prepare a fresh Kimwipe or weigh paper for the next specimen in line. Finally, ensure all tissue is fully submerged in the RNA*later* and place at 4°C for 24 hrs to allow the RNA*later* to infuse the tissue (see RNA*later* specific instructions). After incubating for 24 hrs, spin the microcentrifuge tubes at 2,000 rpm for 1 min to confirm that all legs are submerged and store at -80°C until needed.

### Museum Specimen Curation/ Alcohol Storage

Specimens prepared for long-term museum storage should be kept in 70% isopropanol for two weeks at -20°C in 3 (11.1 mL) or 4 (14.8 mL) dram shell vials (depending on the size of the individual) to allow the alcohol to diffuse into the specimen and reduce brittleness. An initial storage at -20°C for two weeks avoids putrefaction that sometimes results when millipedes are fixed in room-temperature alcohol. After two weeks, alcohol should be replaced and subsequently the material should be stored at room temperature. Depending on the size of the specimen, a second round of isopropanol change may be needed—particularly for large specimens or if the alcohol is darkened to a point where the label is discolored. For museum storage, up to 12 individual shell vials can be stored together in 70% isopropanol kept at room temperature in 473 mL straight-sided glass jars with polypropylene caps and a PTFE cap-liner. The stock caps from individual shell vials should be replaced with a cotton plug (tight enough that the specimen does not fall out if the vial is inverted). This process will ensure diffusion of the isopropanol throughout the jar. Place the vials cotton end down into the jar. Finally, the jar should be topped off with 70% isopropanol. In contrast, field-preserved specimens in ethanol (those which prematurely died in the field) should receive fresh, cold 100% ethanol and remain at -20°C indefinitely.

**Figure 11.**
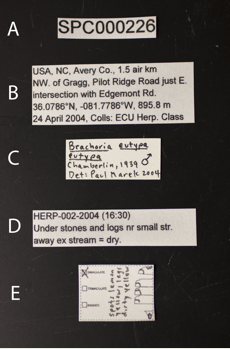
Curatorial labels for alcohol specimens. **A:** Unique specimen code; **B:** Locality, date, collector label; **C:** Determination label; **D:** Habitat and collection code; **E:** Color and pattern label.

Labels prepared on white chemical resistant paper (*e.g.*, Resistall, Forbon, 100% rag content, Tyvek) should be included with each specimen. Each specimen should have five associated labels. (1) Specimen labels should indicate the unique specimen code assigned to each millipede during processing (Fig. 11A). (2) Collection labels include information regarding the country, state/province and county/township of the collection site. This is followed by a short description of location and reference point, GPS coordinates, and elevation. List the collectors, the date and time of the event, and the corresponding collection code. Dates should be designated by day and month in Roman numerals, followed by year (Fig. 11B). (3) Determination labels should include taxonomic identification to species and/or subspecies followed by the authority (Fig. 11C). (4) Follow with a brief summary of environment, ecology, and collection technique (Fig. 11D). (5) Finally, millipede color frequently fades in alcohol and therefore a label explaining the color and pattern of the specimen should be included (Fig. 11E). All labels should be typed using an easy to read sans-serif font, such as Arial, with the unique specimen code large enough for rapid identification (e.g. font size 14). Font size of the other labels will be determined by the amount of information, but font size 6 is typically appropriate.

### Gonopod Dissection and Examination

**Figure 12.**
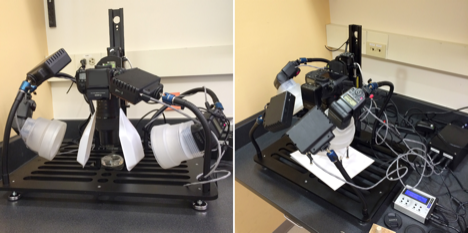
System for photographing the gonopods and other small morphological features of millipedes.

For identification and imaging, the left gonopod should be removed using a thin needle clamped in a pin vise (00 pins or minutens work well). Using the pin, the membrane surrounding the left gonopod should be perforated, and the gonopod completely separated from the membrane. After which, the gonopod should be lifted out using size 55 Inox forceps. Special care must be taken when piercing the membrane between the two gonopods; the membrane is notably tougher in this area and if not properly perforated the right gonopod may accidentally be lifted out with the left. To remove, grasp the inter-gonopodal membrane or the coxal apodeme, taking care to avoid damaging the gonopod. *The actual gonopod should never be grasped itself to remove from the segment*. Dissected gonopods should then be examined to identify the species since they are often the species-characteristic features used for taxonomy. The left gonopod, optionally, can be photographed using a microphotography system (*e.g.*, Canon dSLR with a 65mm macro lens mounted on a Passport II Portable Digital Imaging System, Visionary Digital, Charlottesville, VA, Fig. 12). Gonopods should be photographed from three views: anterior, posterior and medial. Additional angles, such as lateral, should be photographed if they reveal a feature diagnostic for the species. Once photographed, gonopods are placed in 70% isopropanol in glass microvials plugged with cotton and stored with the source material preserved in the 3 or 4 dram shell vials.

## Discussion

The collection and preservation methods presented here are an effective and efficient means of collecting millipedes of the family Xystodesmidae and other terrestrial arthropods, such as centipedes, beetles, and spiders. These methods represent a demonstrably successful model and starting point for investigators interested in collection-based research with most ground-dwelling arthropods, and as such may be modified for other taxa. For example, we have implemented these methods for the collection and preservation of ground beetle specimens and their DNA. Rather than storing specimens in alcohol, however, beetles are pinned and labeled with acid-free 100% cotton archival paper. In addition, these methods can be adapted for individual use and to fit the preexisting workflow of the lab.

Our methodology for hand collecting xystodesmid millipedes was developed to provide a reliable and useful protocol for collection based biodiversity research. Recent studies of millipedes using these techniques and subsequent molecular phylogenetics have shed light on the evolutionary relationships of the Diplopoda, but much remains to be explored solely in terms of an α-taxonomic and primary descriptive standpoint (Marek and Bond 2006, Marek 2010). Despite commitments to slow the rate of species decline, global biodiversity will continue to be lost (Butchart et al. 2010). Collections-based research will therefore become more urgent as rates of habitat and species loss rise, accompanied with the potential for anonymous extinction (a process in which a species is lost before it is discovered, and therefore its role in the ecosystem or potential use for society remains unknown). The benefits of museum collections for education, research and documentation of biodiversity are numerous. For example, extensive biological collections inform conservation strategies that aid in the development of nature preserves and promote the sustainable use of natural resources (Pino-Del-Carpio et al. 2014). Furthermore, exciting and publically-engaging discoveries have been made from previously undescribed museum material, such as *Darwinilus sedarisi*, a species of rove beetle collected by Darwin during the H.M.S. Beagle’s voyage (Chatzimanolis 2014). On average, it takes 21 years for a specimen to be identified and described as a new species, highlighting the need to conserve and support biodiversity collections as active centers of research (Fontaine et al. 2012). In addition, the rise of specimen digitization fosters access to collections, and facilitates species discovery, conservation, and elucidation of evolutionary relationships (Anderson 2012, Cook et al. 2014). Significantly, as mentioned above, anonymous extinction due to habitat destruction necessitates urgency for the discovery and description of biota and raises the grim possibility that much potential advancement in the areas of pharmacology, ecology and genetics will be irreplaceably lost. For these reasons it is imperative that collections based research flourishes and that methods for its conservation and continuity be shared and promoted within the scientific community.

## Supplementary Material

Supplementary material 1 Collection Card Data type: Text
Brief description: An editable Microsoft Word file of the collection card.
File: oo_49783.docx Means, JC; Francis, EA; Lane, AA; Marek, PE

## Figures and Tables

**Figure 1.** Places to collect: lowlands next to stream, stream gully, and old forest road.

**Figure 5.** Use of a millipede collecting rake.

**Figure 7.** Millipede storage in the field.

**Figure 10.** Removing legs for preservation in RNAlater
