# Supplementary material for: A general methodology for collecting and preserving xystodesmid and other large millipedes for biodiversity research
Source: Biodivers Data J. 2015 Aug 17;(3):e5665. doi: 10.3897/BDJ.3.e5665 (PMC4563156; doi:10.3897/BDJ.3.e5665)
Supplement: Supplementary material 1 — Collection Card [file biodiversity_data_journal-3-e5665-s001.docx]

| Collection Code: | State/District: | County/Subdistrict: |
| --- | --- | --- |
| Locality Description (include distance and direction from junction with stream or road): | | Barometric Elevation: |
|  |  | GPS Elevation: |
| Mountain/Mountain Range: | Collecting Method: | GPS Satellite #: |
| Latitude: | Longitude: | GPS Accuracy: |
| GPS Waypoint Name: | Date: | Habitat: |
| Collectors: | Time: |  |
| Notes: | |  |
| Taxa collected: | | |

| Collection Code: | State/District: | County/Subdistrict: |
| --- | --- | --- |
| Locality Description (include distance and direction from junction with stream or road): | | Barometric Elevation: |
|  |  | GPS Elevation: |
| Mountain/Mountain Range: | Collecting Method: | GPS Satellite #: |
| Latitude: | Longitude: | GPS Accuracy: |
| GPS Waypoint Name: | Date: | Habitat: |
| Collectors: | Time: |  |
| Notes: | |  |
| Taxa collected: | | |
